# Immunophenotyping identifies key immune biomarkers for coronary artery disease through machine learning

**DOI:** 10.1371/journal.pone.0328811

**Published:** 2025-08-26

**Authors:** Lelin Jiang, Minghao Jiang, Yiying Liu, Wei Zhao, Xinlang Zhou, Ying Liu, Shue Huang, Lina Chen, Wenbing Jiang

**Affiliations:** 1 Department of Clinical Medicine, The Second Clinical Medical College of Wenzhou Medical University, Wenzhou, Zhejiang, China; 2 Department of Surgery, The Second Hospital of Wenzhou Medical University, Wenzhou, Zhejiang, China; 3 Wenzhou Central Hospital, Wenzhou, Zhejiang, China; 4 Department of Cardiology, The Third Clinical Institute Affiliated to Wenzhou Medical University, Wenzhou, Zhejiang, China; 5 Department of Cardiology, Wenzhou Integrated Traditional Chinese and Western Medicine Hospital, Wenzhou, Zhejiang, China; 6 Department of Cardiology, The Central Affiliated Hospital, Shaoxing University, Shaoxing, Zhejiang, China; Second Xiangya Hospital, CHINA

## Abstract

**Introduction:**

The differences among immune subtypes in coronary artery disease (CAD), their interrelationships, and the associated immune biomarkers remain incompletely understood.

**Methods:**

The samples were collected from the GSE20686 and GSE42148 datasets for analysis. Principal component analysis (PCA) and Gene Set Variation Analysis (GSVA) were performed on the subtypes. Gene Ontology (GO) and Kyoto Encyclopedia of Genes and Genomes (KEGG) analyses were used to determine functional and pathways in CAD. Machine learning models were constructed for CAD prediction. Model validation was performed using GSE56885 and GSE71226 datasets. The expression and function of the identified genes were evaluated using immunohistochemistry, CCK-8 assays, wound healing assays, and Transwell invasion assays.

**Results:**

Multiple immune cells showed correlations with CAD samples. Two immune cell subtypes were identified, with significant differences in programmed cell death-ligand (PD-L1) expression, immune scores, and stromal scores between subtypes (P < 0.05). Three CAD hub genes were identified by WGCNA. GO analysis revealed enrichment in Biological Process (BP) and Molecular Function (MF). Among the several machine learning models, the RF model was selected based on combining parameters. The model mainly included two CAD immune marker genes, AKT1 and PTK2B. Differential expression of AKT1 and PTK2B was observed in cardiac myocytes. Inhibition of PTK2B suppressed cell proliferation and invasion, and induced apoptosis in HUVEC cells.

**Conclusion:**

Immunophenotyping revealed an association between CAD and PD-L1. AKT1 and PTK2B were identified as key disease signature genes, which may hold clinical significance for the diagnosis, prognostic assessment and treatment of CAD.

## Introduction

Coronary artery disease (CAD) is a heart disease caused by myocardial ischemia and hypoxia due to atherosclerosis in the coronary arteries [1], and it is more likely to develop in middle-aged and old individuals [2]. Among cardiovascular diseases, CAD is one of the leading global health concerns [3], with evidences suggesting a hereditary component [4]. Various factors are involved in the diagnosis and management of CAD, and risk factors for the disease can vary across different ethnic populations. Studies have indicated a causal relationship between red blood cell traits and CAD [5]. Early-stage CAD treatment following guideline-recommended pharmacological interventions has been associated with a lower risk of death than conservative treatment, with a median follow-up of 5.7 years [6]. Genetic scoring studies have shown that individuals with high-risk genetic variants, who are three-times more likely to develop CAD, can be identified. However, these individuals cannot be identified solely based on family history or clinical risk factors [7]. Understanding the pathogenesis and developing preventive and therapeutic strategies for CAD is of critical importance. In particular, the role of immune biomarkers in CAD has been receiving increasing attention. However, many aspects of the relationship between immune biomarkers and CAD remain unclear.

While immunotherapy has been widely studied for cancer treatment [8–11], research on its application to non-oncological diseases is ongoing, particularly in myasthenia gravis [12], acute kidney injury [13], and neuromyelitis optica spectrum disorder [14]. In the context of CAD, some immune-related biomarkers have been explored [15–17]. For example, Zhao et al. reported that FBXO7, RAD23A, and MKRN1 have significant association with CD8 + T cells in both CAD and various cancers, suggesting they may play a role in modulating immune responses in these conditions [15]. Yang et al. uncovered that ASCC2, LRRC18, and SLC25A37 can serve as diagnostic indicators for CAD, with immune cell infiltration playing a critical role in the initiation and progression of CAD [17]. Feng et al. developed a diagnostic model for advanced-stage CAD and identified four genes with a strong association with CAD. Notably, IL13RA1 may be involved in the processes of inflammation, fibrosis, and cholesterol efflux in atherosclerosis through the regulation of the JAK1/STAT3 pathway [16]. However, the specific roles and underlying mechanisms of these biomarkers in CAD are largely unknown.

Machine learning models not only provide new avenues for data integration and analysis in biomedicine, but also help identify new biomarkers through prediction algorithms [18–21]. Generalized liner models (GLM) are highly effective when applied to secondary traits [22]. Random forest (RF) uses multiple decision trees for improved prediction accuracy than a single decision tree [23]. Support vector machines (SVM) are well-suited for small sample sizes and nonlinear problems [24], while extreme gradient boosting (XGB) integrates the predictions of multiple classifiers to generate the final result [25]. Machine learning algorithms have become valuable tools for biomarker identification and disease prediction in CAD. In this study, we aim to identify the differences and associations between the different immune subtypes of CAD, explore immune-related biomarkers in CAD and initially explore biological functions and pathways using bioinformatic approaches. Moreover, we utilize immune biomarkers to construct prediction models of CAD. Through the application of machine learning models, biomarkers can be systematically identified and validated, providing new insights into their role and mechanisms in the pathogenesis of CAD.

## Methods and materials

### Data collection and pre-processing

The gene expression profile data used in this study were obtained from the Gene Expression Omnibus (GEO) database [26]. The main datasets analyzed include GSE20686 dataset based on the GPL4133 platform and GSE42148 dataset based on the GPL13607 platform. Two validation datasets, GSE56885 and GSE71226, were also used. All four GEO datasets comprise gene expression profiles from human CAD patients; however, detailed information such as age, gender, and comorbidities is not available. The analysis dataset was created by integrating 393 CAD patient samples from GSE20686, 9 patient samples from GSE42148, and 11 normal samples. As these two datasets were generated from different platforms, the ComBat function was applied to correct for batch effects and minimize platform-related variability.

### Immune cells typing of CAD

The normalized and batch-corrected gene expression matrix was converted into relative proportions of immune cells using the CIBERSORT R script. This method relies on the LM22 signature matrix, which defines gene expression profiles for 22 immune cell subtypes [27]. Immune scores and stromal scores were calculated based on the ESTIMATE algorithm to assess the tumor microenvironment (TME). The correlations between immune cells and these scores were visualized using the “corrplot” package [28]. Immune cell infiltration patterns were further analyzed through consistent clustering using the “ConsensusClusterPlus” package [29], setting the maximum number of clusters to 9, the bootstrap resampling frequency to 50, and sampling 80% of the samples at a time. A fixed random seed was used to ensure reproducibility, and Euclidean distance was chosen as the similarity metric [30]. Differential expression analysis of immune cells and programmed death-ligand 1 (PD-L1) was subsequently performed in the identified clusters.

### Differential genes typing in immune cells of CAD

Differential gene analysis was conducted using a false discovery rate (FDR) < 0.05 and log2 |fold change| ≥ 1 as screening conditions [31] in different subtypes of immune cells. The obtained differential genes were clustered consistently to distinguish subtypes, and the differential expression of PD-L1 and immune cells across subtypes were evaluated.

### Identification of immune-related differential genes

Gene expression profiles of CAD patients and control group were analyzed to identify differentially expressed genes (p < 0.05). Heat maps showing the top 42 highly variable genes were generated using the “ggplot2” package [32]. A list of immune-related genes (IRGs) were obtained from The Immunology Database and Analysis Portal (ImmPort, https://www.immport.org/) [33]. Immune-related differential genes were identified by plotting Venn diagrams to show the intersection between differential genes and immune-related genes. The expression phenomena in different subgroups were analyzed using immune infiltration and immune cell differential analysis.

### Differential IRGs typing of CAD

Differentially expressed IRGs were grouped by consensus clustering analysis. PCA was performed to assess the accuracy of the clustering. A key advantage of PCA over other methods is its ability to preserve essential features of the data while reducing complexity. Moreover, PCA makes fewer assumptions about data distribution, allowing it to perform robustly across a wide range of data types [34]. GSVA analysis was then applied to evaluate transcriptome data for gene set enrichment, highlighting differences in metabolic pathways among the identified subgroups. Compared to other enrichment analysis methods, GSVA is particularly robust in studies with small sample sizes, making it especially suitable for analyzing differential expression of IRGs in CAD [35].

### WGCNA co-expression analysis of IRGs and IRGs typing

Weighted gene co-expression network analysis (WGCNA) is a systems biology method for describing correlation patterns among genes across microarray samples. It enables the identification of clusters (modules) of highly correlated genes, the summarization of these clusters using module eigengene or intramodular hub genes, the exploration of relationships among modules and their associations with external sample traits (through eigengene network methodology), and the calculation of module membership measures [36]. IRG expression data were extracted from the CAD dataset, and the top 20% of the most fluctuating genes were selected for WGCNA analysis [37] to identify the strongest relevant modules for immune-related processes. Using the hierarchical clustering method, the outlier value was set to 20,000 to remove non-standard samples. A scale-free network was constructed using the best power value selected from 20 soft threshold powers. Dynamic tree-based clustering was used to realize co-expression modules and assess module similarity. The most robustly related hub gene modules were selected for subsequent study, with core genes filtered based on gene importance greater than 0.5 and gene-to-module correlation greater than 0.8. The same WGCNA co-expression analysis was performed for IRGs typing to obtain the hub genes of the relevant modules.

### Enrichment analysis of the disease hub genes

The intersection genes from the two related modules identified in cluster WGCNA and disease WGCNA were defined as the hub genes for CAD. GO [38] and KEGG analyses [39] were performed to these CAD hub genes to determine their functions and associated metabolic pathways. GO analysis includes biological process (BP), cellular component (CC) and molecular function (MF). The annotation files for human species were obtained from the “org.Hs.eg.db” package.

### Machine learning model construction and accuracy qualification

Various R packages, including “randomForest”, “kernlaband”, and “xgboost”, were used to build GLM, RF, SVM, and XGB, four types of machine learning models. Residual box plots, cumulative distribution of residuals and ROC curves are used to evaluate the performance of each model, and the best performing model was selected for further analysis. The odds of CAD occurrence in patients were evaluated using a final composite score from a nomogram, which scored the characteristic genes screened by the computerized machine learning algorithm. The nomogram was evaluated by calibration curves and decision curves [40]. This highest performing machine model was validated on two datasets, GSE56885 and GSE71226, for predicting CAD accuracy, with results displayed via ROC curves.

### Immunohistochemistry validation analysis

The Human Protein Atlas (HPA, https://www.proteinatlas.org/) database were used for validation of CAD immune signature genes. Human heart tissue slices from cardiomyocytes were stained with antibodies HPA002891 and HPA026091. The samples included both males and female participants of various ages.

### Cell proliferation, invasion and apoptosis in HUVECs

Human umbilical vein endothelial cells (HUVECs) were purchased from the Cell Bank of the Chinese Academy of Science (Shanghai, China) and cultured in DMEM supplemented with 10% fetal bovine serum at 37°C. Short hairpin RNAs (shRNAs) targeting PTK2B were obtained from GenePharma (Shanghai, China). Transfections were performed using Lipofectamine 3000 reagent (Invitrogen, Carlsbad, USA), and transfection efficiency was assessed by RT-PCR and Western blotting as previously described [41,42]. Primary antibodies used included anti-PTK2B antibodies (CAT# 3480, 1:1000) and anti-GAPDH antibodies (CAT# 2118, 1:5000), both from Cell Signaling Technology (MA, USA). Cell viability was evaluated using the Cell Counting Kit-8 (CCK-8) assay, while cell proliferation was assessed by EdU assays following shPTK2B transfection in HUVECs [43]. Apoptosis was measured using Annexin V-FITC/PI staining, and cell invasion ability was detected using the Transwell chamber invasion assay, as described previously [44].

### Statistical analysis

All statistical analyses were conducted using R software. A p-value < 0.05 was considered statistically significant.

## Results

### Immune cells typing of CAD

**Fig 1:**
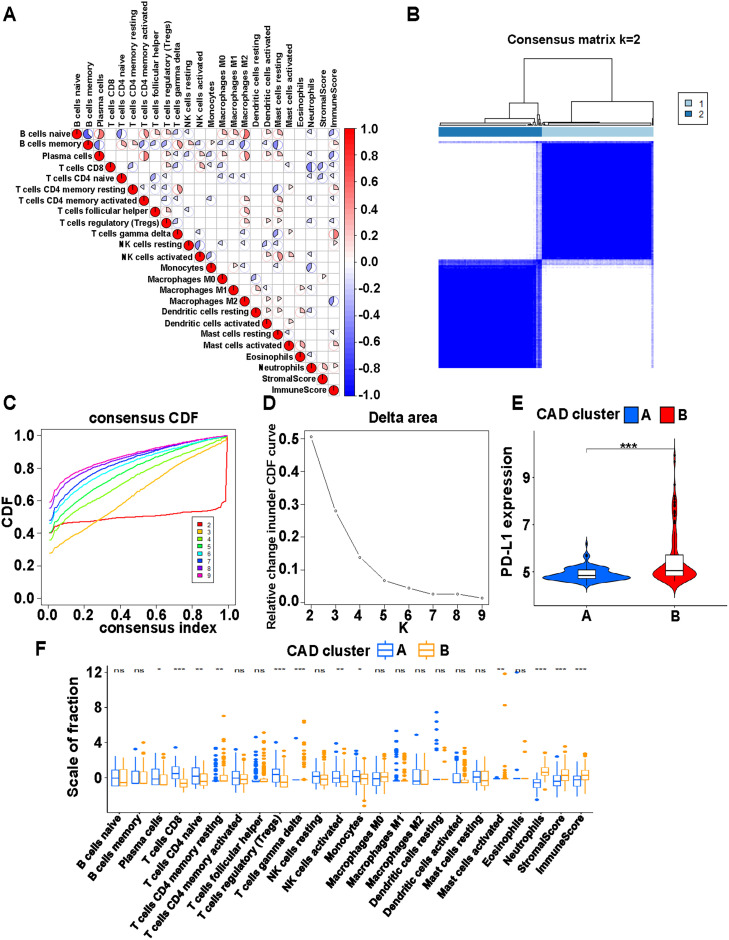
Immune cell typing of CAD. (A) Correlation analysis of 22 types of immune cells, stromal score, and immune score in CAD. Red indicates positive correlation and blue indicates negative correlation. (B) Consensus matrix heat map defining two clusters (k = 2) and their correlation area. (C) Consistency cumulative distribution function (CDF) curve. (D) Delta area curve of consensus clustering in CAD, indicating the relative change in area under the CDF curve for each category number k compared with k-1. The horizontal axis represents the category number k, and the vertical axis shows the relative changes in the area under CDF curve. (E) Differential expression of PD-L1 in different CAD clusters. (F) Immune cell expression differences between CAD cluster A and B. ***p < 0.001, **p < 0.01 and *p < 0.05.

In the immune cell correlation analysis, correlations between 22 immune cells, stromal score, and immune score were visualized (Fig 1A). Correlations between two types of immune cells were highlighted with color: blue for negative correlation, red for positive correlation, with darker colors indicating stronger significance. The consensus matrix for consistent clustering defined two clusters (Fig 1B). The cumulative distribution function curve (Fig 1C) and the delta area curve (Fig 1D) showed that the optimal clustering solution was k = 2. PD-L1 expression was significantly different between the two subgroups (P < 0.001) (Fig 1E). The analysis of differences between immune cells, stromal score, and immune score revealed 12 statistically significant items, including 6 items with P values below 0.001 (Fig 1F).

### Differential genes typing based on immune cell subtypes in CAD

**Fig 2:**
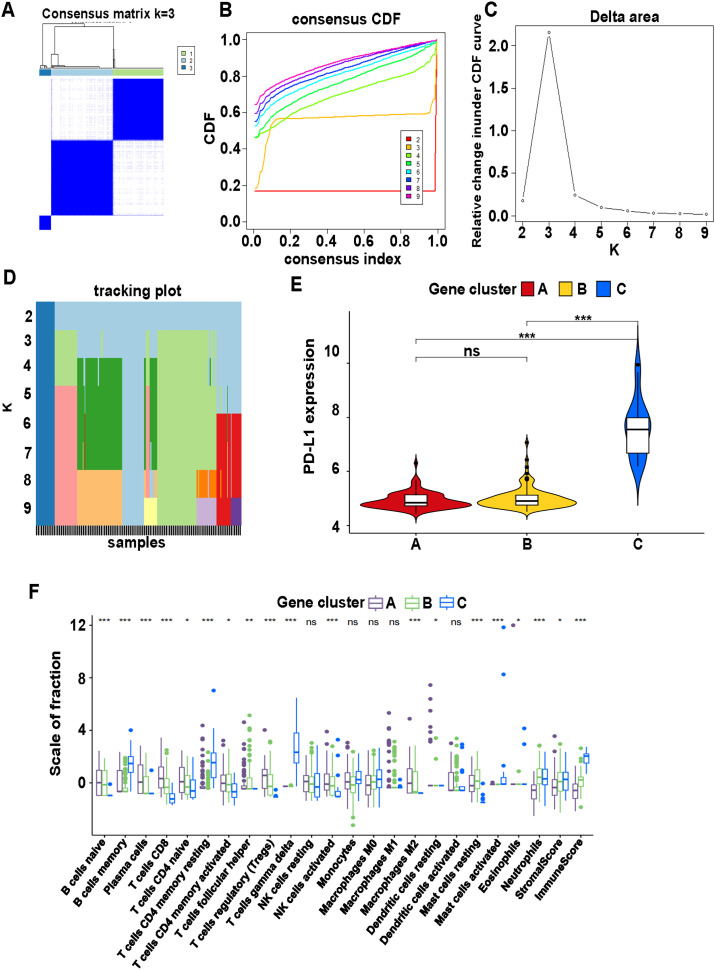
Immune differential gene typing based on different immune cell typing in CAD. (A) Consensus matrix heat map defining three clusters (k = 3) and their correlation area. (B) Consistency CDF curve. (C) Delta area curve of consensus clustering in genes. (D) Tracking plot showing item cluster membership across different k. (E) Differential expression of PD-L1 between gene clusters A, B and C. (F) Immune cells expression differences between gene clusters A, B and C. ***p < 0.001, **p < 0.01 and *p < 0.05.

Based on the two subtypes of immune cells, differential genes in the two subtypes were screened (P < 0.05). The consensus matrix defined three subtype clusters (Fig 2A). Supporting validation graphs, including the concordance CDF function, delta area curves, and K-item tracking plots, confirmed that K = 3 was the optimal number of clusters (Fig 2B–D). The expression of PD-L1 differed significantly among the three genomic subtypes (Fig 2E). Analysis of immune cells revealed significant differences across 13 immune cell types (P < 0.001) (Fig 2F).

### Gene differential expression analysis and identification of immune-related differential genes

**Fig 3:**
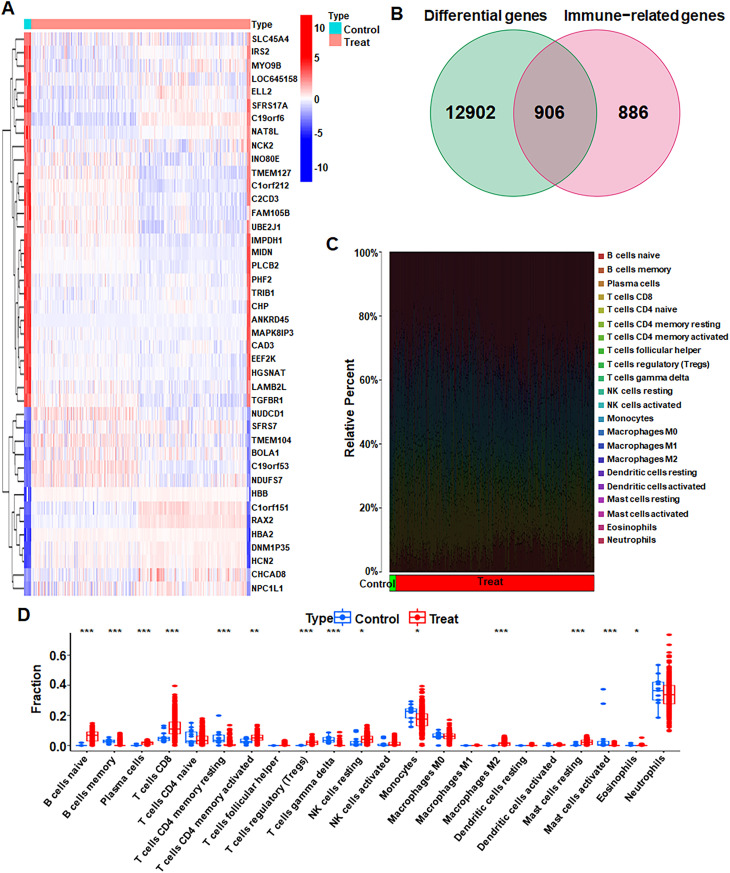
Gene differential expression analysis and immune-related differential gene identification. (A) Heat map of differentially expressed genes in CAD. Red represents positive correlation, and blue represents negative correlation. (B) Venn diagram showing intersecting genes between differential genes and immune−related genes. (C) Immune cell types and their ratios in CAD patients. (D) Differentiation of 22 immune cell types and immune score in control and treatment subgroups.

Analysis of gene expression in CAD samples from the treatment and control groups revealed 13,808 differentially expressed genes. A heat map shows the top 42 differential genes, with 28 genes positively correlated and 14 genes negatively correlated in the control group (Fig 3A). In the treatment group, most genes were negatively correlated. A list of 1,792 IRGs was downloaded from the online tool website, and a Venn diagram identified 906 intersecting genes (Fig 3B). Immune infiltration results indicated higher B cells naive content in the treatment group (Fig 3C). Variability were observed across 14 immune cells such as Mast cells activated, Mast cells resting, and Macrophages M2 (Fig 3D).

### Differential IRGs typing of CAD

**Fig 4:**
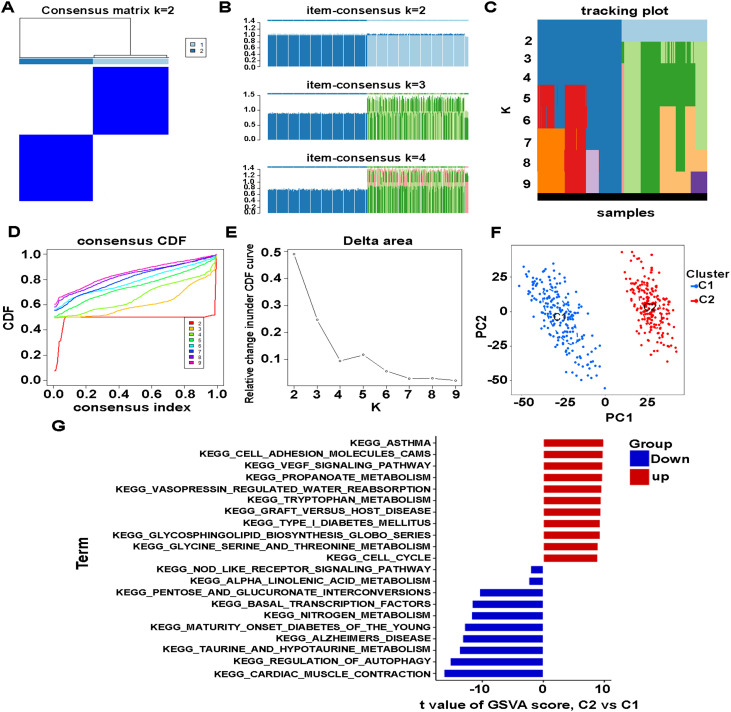
Differential Immune-related genes typing of CAD. (A) Consensus matrix heat map defining two clusters (k = 2) and their correlation area. (B) Item-consensus plot. (C) Tracking plot showing item cluster membership across different k values. (D) Consistency CDF curve. (E) Delta area curve of consensus clustering. (F) PCA analysis classifying immune genotyping between C1 and C2. (G) GSVA of biological pathways between two distinct subtypes, where red and blue represent up-regulated and down-regulated pathways, respectively.

Consistency clustering analysis of 906 differential IRGs defined two clusters (Fig 4A). The Item-consensus plot supported K = 2 as the optimal clustering solution (Fig 4B). Additional validation plots confirmed this result (Fig 4C–E). PCA analysis clearly distinguished C1 and C2 subtypes, indicating effective clustering (Fig 4F). GSVA analysis revealed significant difference in pathways between C1 and C2 subgroups (Fig 4G). Among the 11 pathways in the C1 subtype, “cardiac muscle contraction” was the most enriched pathway. Among the 11 pathways in the C2 subtype, “asthma”, “cell adhesion molecules cams”, “vegf signaling pathway” and “propanoate metabolism” all have the same number of genes enriched.

### WGCNA co-expression analysis of IRGs expression

**Fig 5:**
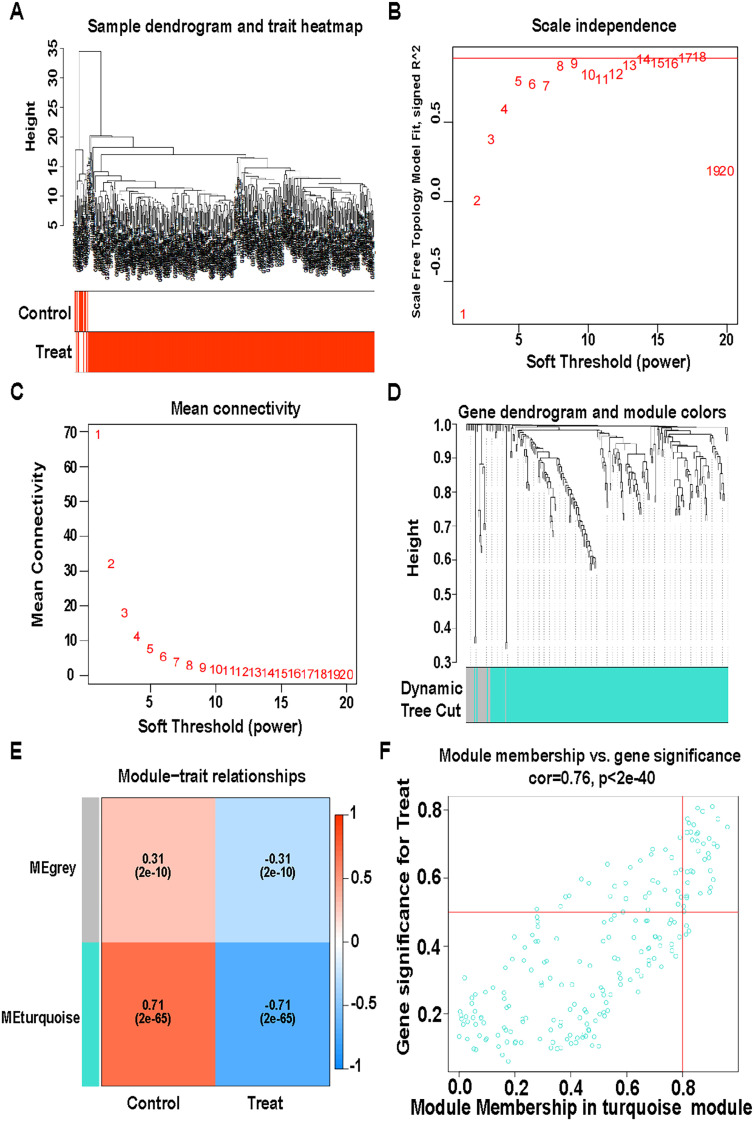
WGCNA co-expression analysis of immune genes. (A) Sample dendrogram and trait heatmap. (B) Distribution of the scale-free topology model fit. (C) Trends of mean connectivity. (D) Hierarchical clustering analysis showing similar characteristics in a dendrogram with the same colors. (E) Pearson correlation analysis of merged modules and CAF scores. (F) Scatterplot of module membership (MM) and gene significance (GS) from the turquoise module.

A sample dendrogram and trait heatmap were generated for CAD samples (Fig 5A). A weighted value of 0.9 ensured consistency in the scale-free network (Fig 5B, C) [45]. Two functional modules were identified, including the grey module (19 genes) and the turquoise module (208 genes) (Fig 5D). Thirty-five turquoise module hub genes were screened based on gene importance and gene to module correlation. The turquoise module showed the strongest association with CAD (R = 0.71, p = 2e-65) (Fig 5E), and turquoise module membership and gene significance were highly correlated (cor = 0.76, p < 2e-40) (Fig 5F).

### Co-expression analysis of WGCNA based on IRG subtypes

**Fig 6:**
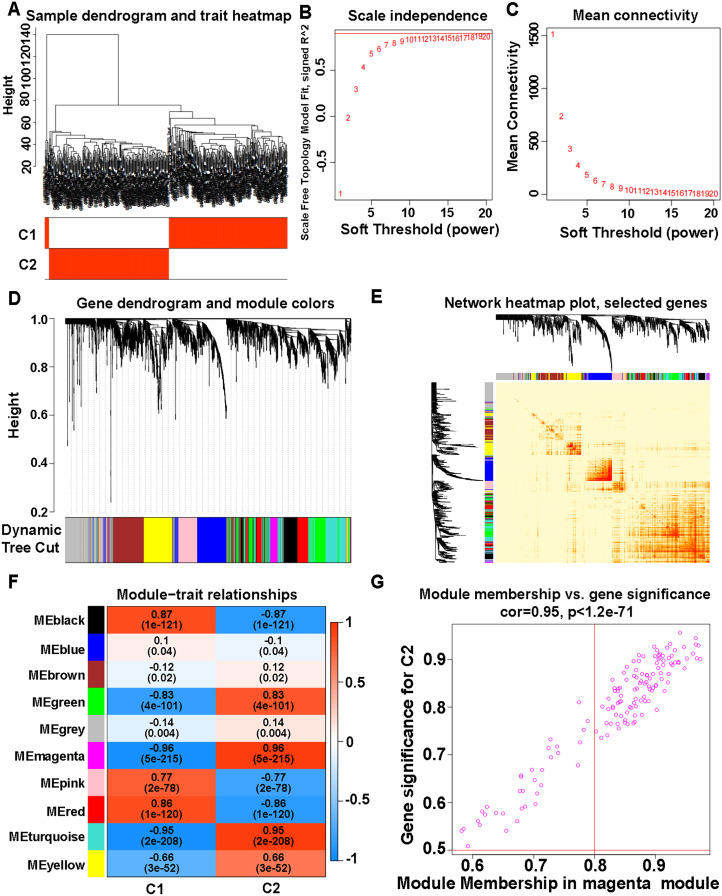
Co-expression analysis of WGCNA based on immune gene typing results. (A) Sample dendrogram and trait heatmap. (B) Distribution of the scale-free topology model fit. (C) Trends of mean connectivity. (D) Hierarchical clustering analysis showing similar characteristics in a dendrogram with the same color. (E) Network heatmap plot of selected genes. (F) Module−trait relationships. (G) Scatterplot of MM and GS from the magenta module.

Samples were distributed between the C1 and C2 subtypes (Fig 6A). A scale-free network showed good agreement for setting weighting values (Fig 6B, C). A total of ten modules were obtained (Fig 6D), including black (355 genes), blue (574 genes), brown (506 genes), green (432 genes), grey (647 genes), magenta (140 genes), pink (338 genes), red (419 genes), turquoise (681 genes), and yellow (486 genes) modules. Nine modules were further screened to obtain hub genes, including black (681 genes), blue (5 genes), brown (3 genes), green (559 genes), magenta (588 genes), pink (245 genes), red (638 genes), and turquoise (944 genes), and yellow (193 genes) modules. The network heatmap plot displayed the correlation between selected genes in different modules (Fig 6E). The magenta module had the strongest correlation (R = 0.96, P = 5e-215) (Fig 6F), and the scatterplot of MM and GS in the magenta module confirmed this result (Fig 6G).

### Enrichment analysis of the 3 CAD hub genes

**Fig 7:**
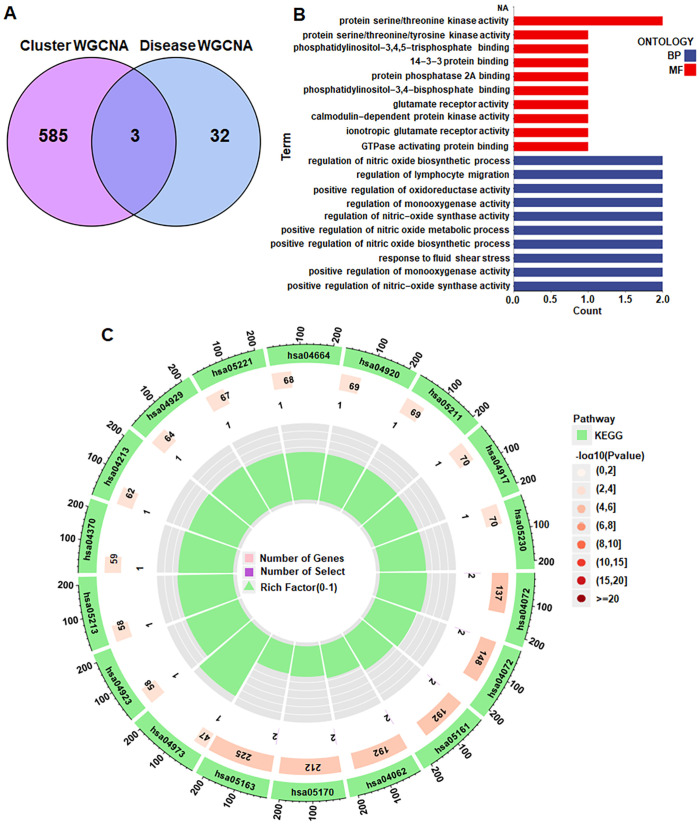
Enrichment analysis of the 3 disease hub genes. (A) Venn diagram showing the intersecting hub genes from Cluster WGCNA and Disease WGCNA. (B) GO analysis. (C) KEGG analysis.

From the disease WGCNA turquoise module, a total of 35 hub genes were obtained, while 588 hub genes were identified from the cluster WGCNA magenta module. Three genes, including AGER (advanced glycosylation end-product receptor), PTK2B (protein tyrosine 2 beta), and AKT1, were obtained at the intersection (Fig 7A). GO analysis (Fig 7B) revealed enrichment in two items, BP and MF. The most enriched function in MF is “protein serine/threonine kinase activity”, while other pathways showing single-gene enrichment. In BP, all 10 pathways were enriched with two genes, with half positively regulating functions, including “positive regulation of oxidoreductase activity”, “positive regulation of nitric oxide metabolic process”, “positive regulation of nitric oxide biosynthetic process”, “positive regulation of monooxygenase activity”, and “positive regulation of nitric-oxide synthase activity”. KEGG analysis (Fig 7C) highlighted six major enrichment pathways, namely “Yersinia infection”, “Phospholipase D signaling pathway”, “Hepatitis B”, “Chemokine signaling pathway”, “Human immunodeficiency virus 1 infection”, and “Human cytomegalovirus infection”.

### Machine learning model construction

**Fig 8:**
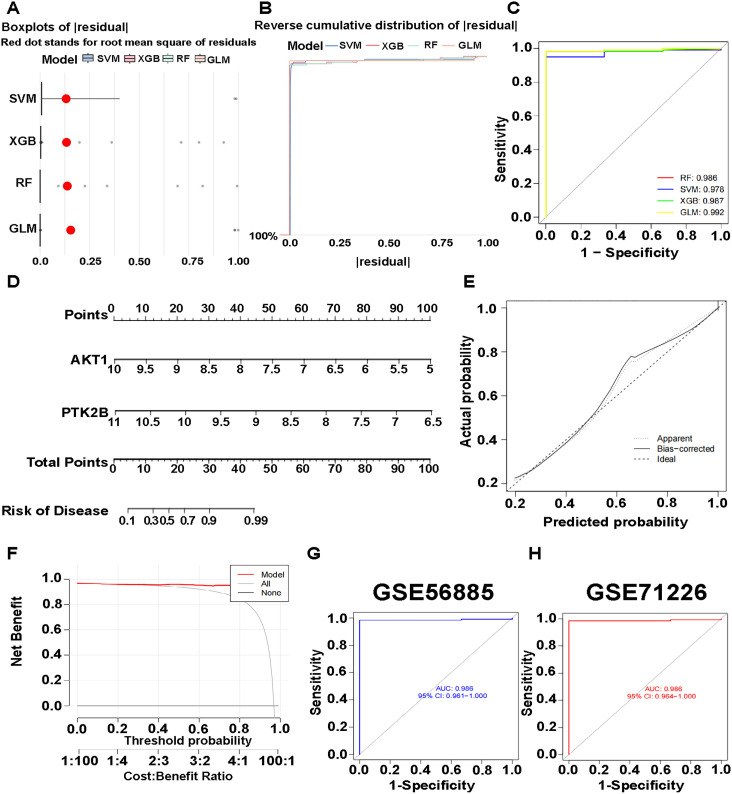
Machine learning model construction and accuracy qualification. (A) Residual box line diagram of four machine learning algorithms. (B) Reverse cumulative distribution of residual among different machine learning models. (C) ROC curve validating the accuracy of the model. (D) Nomogram showing the prediction of CAD prevalence using signature genes. (E) Calibration curve illustrating the accuracy of nomogram. (F) Decision curve illustrating the accuracy of the model. (G) Validation of model accuracy on the GSE56885 dataset. (H) Validation of model accuracy on the GSE71226 dataset.

Four machine learning models were constructed to predict CAD using the three CAD hub genes. The residual box line plot (Fig 8A) displayed the residual values of the machine learning models, while the residual reverse cumulative distribution plot further validated the residual values (Fig 8B). The area under the ROC curve (Fig 8C) confirmed the predictive power of the model, with larger areas under the curve indicating better accurate prediction.

### Machine learning model prediction accuracy

The three diagrams described above were constructed to evaluate the performance of the machine learning model RF. A nomogram scored AKT1 and PTK2B genes, allowing the assessment of CAD risk (Fig 8D). The calibration curve compared the prediction accuracy to actual outcomes (Fig 8E). The decision curve showed that the RF has better predictive ability, as it deviated from the none curve (Fig 8F). The prediction accuracy of the RF model was 98.6% in both GSE56885 and GSE71226 datasets, validated for CAD (Fig 8G, H).

### Immunohistochemistry validation

**Fig 9:**
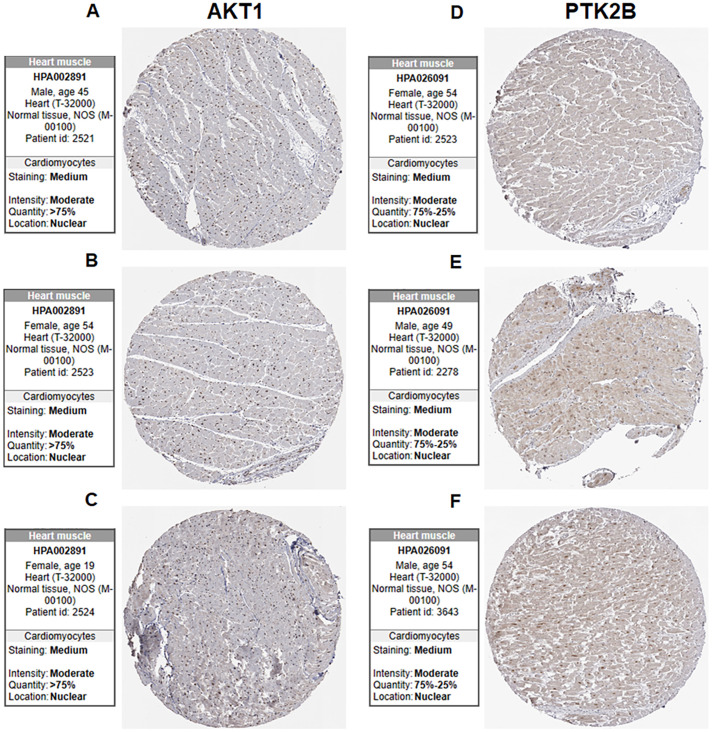
Immunohistochemistry confirming the differential expression of AKT1 and PTK2B in heart muscle.

For the AKT1 gene, we selected samples of male (age 45), female (age 54) and female (age 19). The nuclear intensity of these samples was moderate, with quantity in over 75% of cells (Fig 8A–C). In contrast, for the PTK2B genes, samples from female (age 54), male (age 49), male (age 54), showed moderate nuclear intensity, but quantity in 25% to 75% of cells (Fig 9D–F). This suggests that the expression of AKT1 gene is higher than PTK2B expression in cardiomyocytes.

### Downregulation of PTK2B suppresses proliferation and invasion of HUVECs

**Fig 10:**
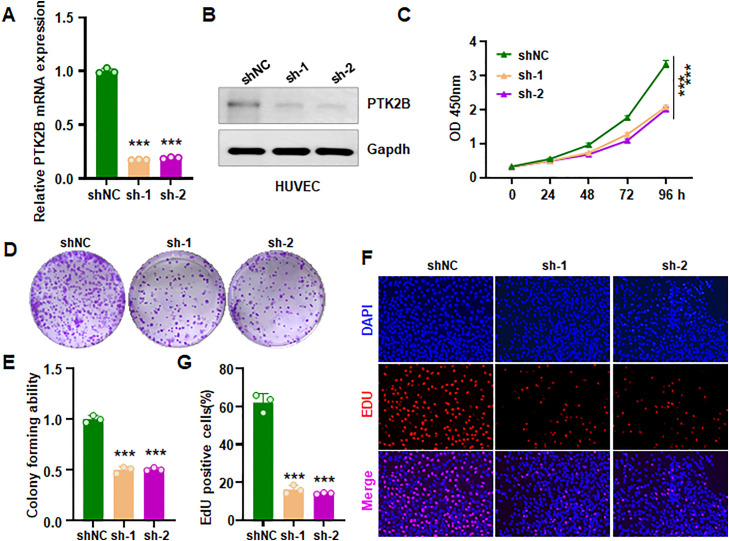
PTK2B knockdown inhibits the viability and proliferation of HUVECs. (A) RT-PCR analysis showing reduced mRNA levels following shPTK2B transfection in HUVECs. shNC: shRNA negative control; sh-1: shPTK2B-1; sh-2: shPTK2B-2. (B) Western blot analysis confirming decreased PTK2B protein levels after shPTK2B transfection. (C) CCK-8 assay demonstrating reduced cell viability in HUVECs following PTK2B knockdown. (D) Colony formation assay showing impaired colony-forming ability after PTK2B knockdown. (E) Quantification of colony formation results. (F) EdU assay indicating decreased proliferation of HUVECs upon PTK2B knockdown. (G) Quantification of EdU-positive cells in HUVECs.

**Fig 11:**
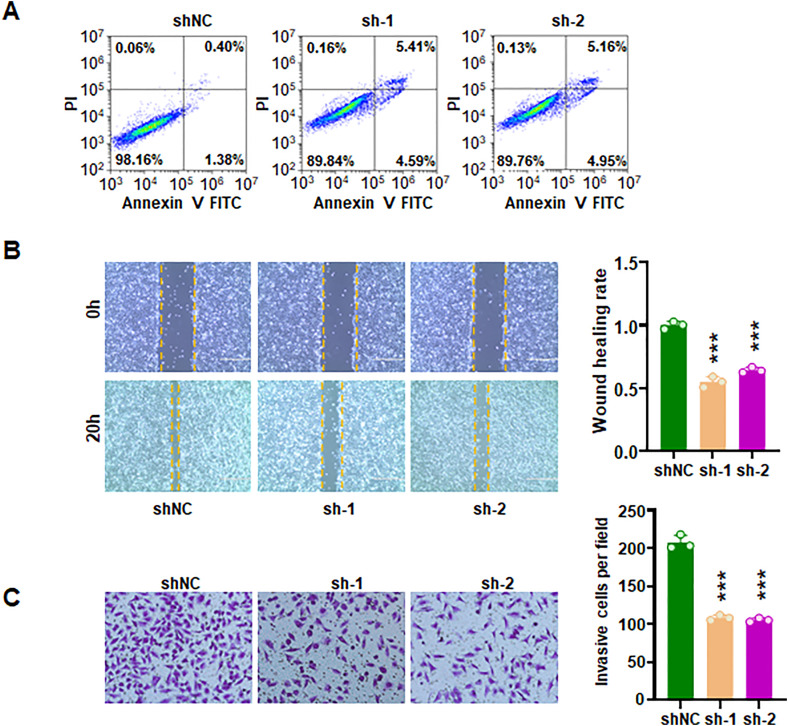
PTK2B knockdown triggers apoptosis and inhibits invasion in HUVECs. (A) Annexin V-FITC/PI staining showing that shPTK2B triggers cell apoptosis in HUVECs. (B) Left: Wound healing assay demonstrating reduced cell migration following PTK2B knockdown. Right: Quantification of migratory distance. (C) Left: Transwell invasion assay showing decreased invasive ability of HUVECs after PTK2B knockdown. Right: Quantification of invaded cells.

To determine the role of PTK2B in HUVECs, shPTK2B was transfected into HUVECs. RT-PCR and Western blotting assays confirmed that shPTK2B reduced both mRNA and protein levels of PTK2B in HUVECs (Fig 10A–B). PTK2B inhibition led to a decrease in cell viability in HUVECs (Fig 10C). Moreover, suppression of PTK2B reduced colony formation in HUVECs (Fig 10D–E). Consistently, EdU incorporation assays revealed that shPTK2B reduced proliferation of HUVECs (Fig 10F–G). In parallel, shPTK2B transfection triggered apoptosis in HUVECs (Fig 11A). Wound healing assays demonstrated impaired cell migration following PTK2B knockdown (Fig 11B). Notably, Transwell assay further revealed that shPTK2B reduced the invasive ability of HUVECs (Fig 11C). Collectively, these results indicate that PTK2B downregulation inhibits the proliferation, migration and invasion of HUVECs.

## Discussion

PD-L1 has demonstrated promising clinical efficacy in immunotherapy across various tumor types [46]. Its sustained expression in tumor cells is critical for promoting tumor immune escape and inducing host T-cell dysfunction [47]. Although the role of PD-L1 in tumor immunotherapy has been extensively studied, its involved in cardiovascular disease, particularly CAD, remains an emerging area of research. Notably, studies have reported that the PD-1/PD-L1 axis play a crucial role in vascular inflammatory diseases [48,49]. Moreover, PD-1/PD-L1 pathway has been verified to regulate proatherogenic T cell immunity via modulating pro-inflammatory and anti-inflammatory cytokine production in CAD [50]. In this study, both immune cell profiling and differential genotyping in immune cell subgroups revealed significant differences in PD-L1 expression in CAD patients. KEGG pathway analysis identified the “PD-L1 expression and PD-1 checkpoint pathway in cancer”, highlighting the potential role of PD-L1 in CAD immunotherapy. This finding is important, as it suggests that PD-L1 may serve as a novel therapeutic target in CAD and could open new avenues for PD-L1-based immunotherapeutic strategies. Additionally, stromal and immune scores varied significantly among immune subtypes, further underscoring the critical role of immune cells in CAD pathogenesis. These results reveal that immune cell-based subtyping may offer new strategies for the personalized treatment of CAD in the future.

In the comparison of IRG subtypes between control and treatment groups, GSVA results for the C1 subtype showed enrichment in pathways related to “cardiac muscle contraction” and “regulation of autophagy”. Meanwhile, the C2 subtype had five enriched pathways. Combining targeted cellular autophagy and immunotherapy could help counteract the drug resistance of the disease [51]. Autophagy-mediated expression clusters have been reported to play a critical role in regulating immunity in CAD [52]. One study identified that autophagy could be a downstream of telomerase and is responsible for CAD pathological switch [53]. Three key genes, including AGER, PTK2B and AKT1, were identified through the intersection of cluster WGCNA and disease WGCNA. One study has shown that receptor for AGER in subcutaneous adipose tissue is associated with CAD development [54]. Another study demonstrated that four genetic polymorphisms of AGER is correlated with CAD occurrence [55]. Among MF categories, “protein serine/threonine kinase activity” was the most significantly enriched function. RIPK1 (receptor-interacting serine/threonine-protein kinase 1) has been identified as a core driver of CAD, activating pathways that promote the release of inflammatory cytokines [56].

The RF model, selected as the good-performing machine learning algorithm, identified two critical immune-related signature genes for CAD: AKT1 and PTK2B. Previous studies have highlighted the important of these genes in cardiovascular disease. Shang et al. reported that FAL1 activates the PTEN/AKT pathway and promotes endothelial cell proliferation in CAD [57]. Luo et al. demonstrated that the AKT pathway is involved in deregulation of ADTRR and LDLR/CD36/LOX-1 signaling, contributing to CAD [58]. Evidence from 2007 indicated that AKT1 deletion exacerbates atherosclerosis and occlusive CAD, as genetic ablation of AKT1 on an apolipoprotein E knockout background (ApoE(-/-)AKT1(-/-)) led to increased aortic lesion expansion and coronary atherosclerosis [59]. Furthermore, loss of AKT1 attenuated the survival and migration of vascular smooth muscle cells and caused cardiac dysfunction in the setting of atherosclerosis [60]. AKT1 expression in vascular smooth muscle cells was also found to influence plaque vulnerability, thereby affecting atherosclerosis progression [61]. PTK2B has been implicated in cell growth, inflammatory responses, and vascular contraction, and has been associated with hypertension [62]. Additionally, PTK2B expression has been linked to ischemic cardiomyopathy [63]. Another study revealed that PTK2B expression was related to vascular calcification via interaction with the ASS1 protein [64]. In this study, we observed that PTK2B regulated proliferation, migration and invasion of HUVECs. Together, these findings indicate that AKT1 and PTK2B could play essential roles in the development and progression of CAD.

## Conclusions

In this study, we revealed an association between CAD and PD-L1. AKT1 and PTK2B were identified as key disease signature genes and demonstrated high predictive accuracy for CAD. While PTK2B has been studies extensively in Alzheimer’s disease [65–67], its role in CAD remains unexplored, suggesting that it could be a new biomarker for CAD treatment and prevention. It is worthy to note that there are several limitations in our study. In vivo experiments are required to determine whether AGER, AKT1 and PTK2B are involved in CAD development. Moreover, IHC experiments are necessary in CAD tissues to validate whether AKT1 and PTK2B are biomarkers for predictive accuracy for CAD. Furthermore, in future studies, we plan to investigate the specific mechanisms by which PD-L1 contributes to CAD and explore whether targeting the PD-1/PD-L1 axis could serve as an effective strategy. This will involve additional preclinical research and clinical trial validation. Beyond PD-L1, we will also examine the roles of other immune biomarkers in CAD to assess their potential as therapeutic targets or diagnostic biomarkers. Through continue research, we aim to deepen our understanding of these immune biomarkers and to develop more effective strategies for the diagnosis and treatment of CAD. Additionally, we intend to explore the potential of combination therapies, such as integrating drug therapy, immunotherapy, gene therapy, and other approaches, to enhance treatment outcomes. By studying combination strategies, we hope to provide more personalized and effective treatment option for patients with CAD.

This study is based on the secondary use of GEO data, which introduces potential limitations related to data quality and sampling characteristics. First, the quality and completeness of the data may vary depending on the original studies, potentially affecting the reliability and accuracy of our analysis. Second, sampling bias may exist, as the datasets are derived from different study populations with potentially inconsistent patient selection criteria, disease stage, and treatment histories. Additionally, the lack of detailed clinical information (e.g., age, gender, comorbidities) limits our ability to perform in-depth analyses of the associations between gene expression and clinical variables. Therefore, although GEO databases provide a valuable resource for large-scale research, it is essential to recognize these limitations and interpret the findings with appropriate caution. In future studies, we plan to conduct mechanistic investigations using CAD tissue specimens and multi-lineage cellular models to further elucidate the underlying pathogenic pathways. Additionally, we intend to perform IHC validation and longitudinal outcome assessments using multicenter clinical cohorts to strengthen the translational relevance of our findings.

## Supporting information

S1 Fig Protein expression of PTK2B in HUVECs after shPTK2B transfection detected by western blotting. (PDF)

S1 Dataset Dataset for Fig 10A. (XLS)

S2 Dataset Dataset for Fig 10C. (XLS)

S3 Dataset Dataset for Fig 10E. (XLS)

S4 Dataset Dataset for Fig 10G. (XLS)

S5 Dataset Dataset for Fig 11B. (XLS)

S6 Dataset Dataset for Fig 11C. (XLS)
