# Supplementary figures and images for: Immunophenotyping identifies key immune biomarkers for coronary artery disease through machine learning
Source: PLoS One. 2025 Aug 26;20(8):e0328811. doi: 10.1371/journal.pone.0328811 (PMC12380355; doi:10.1371/journal.pone.0328811)

PTK2B

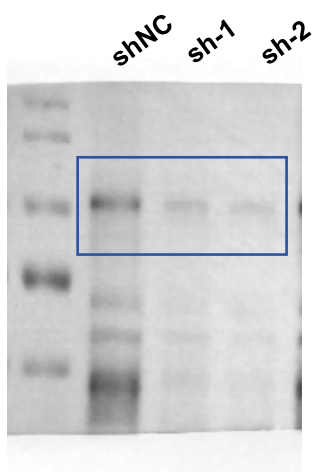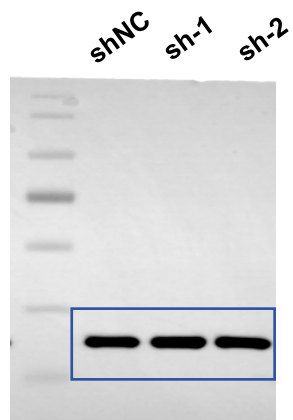

GAPDH

Supplement: S1 Fig — (PDF) [file pone.0328811.s001.pdf]
